# Generative AI in education: Process-aware pedagogy, assessment integrity, and institutional governance

**DOI:** 10.12688/openreseurope.23343.1

**Published:** 2026-05-13

**Authors:** Nora Pireci Sejdiu, Simone Grassini, Sejdi Sejdiu

**Affiliations:** 1 Department of Mathematics, University of Prishtina 'Hasan Prishtina', Prishtina, Prishtina, Kosovo (Serbia and Montenegro); 2 Department of Psychosocial Science, University of Berge, Bergen, Hordaland, Norway; 3 Department of English Language and Literature, University of Prizren, Prizren, Prizren, Serbia

**Keywords:** Generative Artificial Intelligence, ChatGPT, Higher Education, Academic Integrity, AI Ethics, Assessment Practices, Institutional Policy

## Abstract

**Background:**

Generative artificial intelligence (GenAI), particularly large language models such as ChatGPT, is rapidly transforming higher education by reshaping how knowledge is produced, assessed, and mediated through digital systems. While these tools offer new opportunities for learning support and instructional efficiency, they also raise significant concerns regarding academic integrity, institutional governance, and ethical sustainability.

**Methods:**

This study conducts a qualitative thematic literature review of research published between 2020 and 2025. Using thematic synthesis, the study analyzes scholarly articles, institutional reports, and policy discussions addressing the integration of generative AI in higher education. The analysis focuses on four thematic areas: the rise of GenAI in educational contexts, academic integrity challenges, institutional and policy responses, and ethical and environmental implications.

**Results:**

The review identifies four key tensions shaping current debates: rapid student adoption outpacing institutional readiness; growing ambiguity in authorship and assessment practices; fragmented policy responses ranging from prohibition to cautious integration; and limited attention to the ethical and environmental costs of large-scale AI deployment. The findings indicate that these issues are frequently discussed in isolation rather than as interconnected challenges.

**Conclusions:**

The study argues for a process-aware pedagogical framework, referred to as augmented pedagogy, that positions generative AI as a cognitive scaffold rather than a substitute for learning. Such an approach emphasizes transparency in AI use, process-oriented assessment, and responsible institutional governance to ensure that AI integration strengthens rather than undermines the educational mission of universities.

## Introduction

The meteoric advancement of Generative Artificial Intelligence (GenAI) technologies has profoundly altered the landscape of higher education, prompting urgent discussions about the future of teaching, learning, and academic integrity. Among these technologies, ChatGPT has emerged as a leading tool, generating human-like text and providing users with highly coherent responses to complex prompts. Its widespread adoption has been especially prominent among students, raising critical questions about its pedagogical implications and ethical use.

This growing dependence on AI tools by students has created a technological divide within academia. While digital-native students increasingly incorporate AI into their academic routines, many university instructors, particularly those unfamiliar with emerging technologies or lacking strong English proficiency, struggle to keep pace. ^22^ As a result, there is a widening gap between the ways students and faculty engage with digital tools, underscoring the need for systemic adaptation and digital literacy among all stakeholders.

Recent work has highlighted the subtle yet transformative impact of generative AI on higher education, particularly in reshaping assessment practices, digital literacies, and institutional responses to emerging ethical challenges. ^37^

One of the most pressing challenges relates to academic integrity. Students can now use ChatGPT to generate entire essays and assignments, often undetectable by traditional plagiarism detection software. ^43, 26^ This development complicates assessment practices and forces educators to reconsider the reliability of written assignments as indicators of student learning. It also raises fundamental concerns about whether assessments reflect students’ actual knowledge or simply their competence in using AI tools. ^1^ Educators now face the dilemma of determining whether they are grading the student, or the AI.

In response, higher education institutions have adopted varied strategies. Some, such as the New York City Education Department, have implemented outright bans on ChatGPT, blocking access on school networks and devices. ^13^ Prestigious European institutions like Sciences Po have warned students of severe consequences, including expulsion, for unauthorized use of AI in academic work. ^16^ In contrast, others have embraced the technology by issuing guidelines for its ethical use and exploring ways to integrate it into curricula. ^36^ The lack of a coordinated response illustrates a policy vacuum, with many universities still struggling to develop clear, context-sensitive frameworks to guide responsible AI use.

Despite the concerns, GenAI tools also present transformative potential. Recent bibliometric mapping of generative AI research in higher education confirms both the rapid expansion of the field and its thematic fragmentation, reinforcing the need for integrative reviews that connect pedagogical, integrity, policy, and ethical perspectives. ^11^ Scholars argue that these tools can enhance learning by supporting multilingual access, offering real-time feedback, and helping educators tailor content to individual student needs. ^2, 9, 17^ At the same time, they can improve administrative efficiency, assist in grading, and support faculty in curriculum development. ^1^ However, this transformation is accompanied by ethical and environmental implications. The reliance of GenAI systems on massive data processing brings significant risks related to data privacy, algorithmic bias, and the carbon footprint associated with training large models. ^7, 18, 31^

Moreover, the overuse of AI tools in academia may unintentionally diminish critical thinking and problem-solving skills among students, particularly if such technologies are used to circumvent genuine cognitive effort. ^24, 29^ Educators like Victoria Livingstone have voiced concerns that the growing use of AI in writing assignments undermines students’ intellectual development and bypasses the rigorous mental processes associated with academic writing. ^28^ This concern emphasizes the need to preserve the human dimension of education, particularly the teacher’s role in fostering independent thought, ethical reasoning, and disciplinary mastery.

While recent research offers important insights into the implications of AI in higher education, most studies tend to address specific issues, such as cheating, assessment reform, or ethical use, in isolation. There is a pressing need for a more integrative understanding of how AI is reshaping higher education across pedagogical, policy, ethical, and environmental dimensions.

This study addresses that need by conducting a thematic literature review to synthesize current academic and institutional perspectives on the integration of generative AI in higher education. It aims to identify dominant concerns, policy gaps, and best practices, ultimately proposing a balanced framework for responsible and equitable AI adoption in universities.

### Literature review

The introduction of GenAI tools such as ChatGPT has led to a growing body of literature examining the implications of AI integration in higher education. Much of this work explores not only the potential benefits of GenAI but also the deep ethical, pedagogical, and policy-related concerns it raises. This review synthesizes existing research across four key thematic areas: the rise of GenAI in educational contexts, its impact on academic integrity and learning processes, institutional responses and policy developments, and the ethical and environmental considerations surrounding its use.

### The rise of generative AI in higher education

Generative AI has rapidly emerged as a transformative force in academia, capable of producing human-like content in response to diverse and complex prompts, defining GenAI as a deep-learning-based technology designed to generate content, ranging from text to multimedia, based on extensive training data. ^26^ ChatGPT, one of the most prominent examples, has grown to over 300 million weekly users, illustrating the scale of its adoption and its appeal among students seeking efficiency in academic work. ^44^

Several scholars have pointed out that AI tools like ChatGPT are increasingly being used to support a variety of academic tasks, including writing assistance, translation, personalized feedback, and even administrative support. ^3, 30^ These technologies are also being praised for their capacity to personalize learning by offering flexible, student-centered experiences. ^10^ Additionally, researchers have explored the potential of GenAI to aid educators in content development, coding, and communication with students, contributing to a redefinition of faculty roles. ^10, 17^

### Academic integrity and the changing nature of learning

The literature consistently underscores the tension between technological innovation and academic integrity. Recent studies report that students are increasingly turning to GenAI to complete academic assignments, often producing outputs that current plagiarism detection software struggles to flag. ^43, 26^ This raises fundamental questions about the reliability of traditional assessment methods and the extent to which student work reflects genuine learning or strategic AI usage. Recent systematic reviews confirm that generative AI exacerbates academic integrity challenges by complicating authorship and enabling surface-level performance that may obscure limited cognitive engagement, further underscoring the need for assessment redesign rather than reliance on detection-based controls. ^4^

This shift has alarmed educators and researchers alike, who highlight the growing difficulty in determining whether educators are assessing student knowledge or their ability to use ChatGPT effectively. ^1^ Concerns about the erosion of critical thinking skills have also been raised, ^24, 29^ particularly when AI is used to circumvent the writing and analytical processes that are central to deep learning.

Recent research has begun to systematize assessment responses to generative AI through the identification of so-called transparency mechanisms, including structured AI-use disclosures, process documentation, reflective statements, and staged drafting. A review of emerging practices highlights how such mechanisms reorient academic integrity away from detection toward making learning processes visible, while also noting practical constraints related to workload, monitoring, and student acceptance. This strand of research signals a growing interest in pedagogical approaches to integrity, while leaving open questions regarding scalability and disciplinary applicability. ^32^

The anecdotal evidence shared by Livingstone further illustrates this concern. Her decision to leave teaching was driven by the realization that AI-generated work was replacing student thinking. For her, writing was more than a product; it was a cognitive and reflective act that could not simply be outsourced to machines. Such testimonies highlight the broader pedagogical risks associated with unchecked AI adoption in academic environments. ^28^

### Institutional and policy responses

Universities worldwide have adopted varying and sometimes contradictory stances on the integration of GenAI. Some institutions, such as California State University, have taken proactive steps by collaborating with OpenAI to integrate ChatGPT into educational workflows. ^33^ Their approach reflects optimism about AI’s potential to enhance tutoring, personalize learning, and streamline administrative processes.

Conversely, other institutions have reacted with skepticism and caution. For example, the New York City Education Department has blocked access to ChatGPT on school devices, while France’s prestigious Sciences Po warned students of severe penalties for its unauthorized use. ^13, 16^ Between these extremes, many universities are still navigating uncharted territory, struggling to define when and how AI tools should be used. The absence of unified institutional frameworks or clear ethical guidelines has created ambiguity for faculty and students alike. ^40^

This disparity in policy responses reflects a broader uncertainty about how to integrate GenAI into existing educational systems without compromising academic standards and emphasizes the importance of equipping students with guidance rather than simply imposing bans, suggesting a more nuanced, education-driven approach to policy design. ^36^

### Ethical and environmental implications

Beyond pedagogy and policy, the integration of AI into education raises pressing ethical and environmental questions. A major area of concern is the privacy risk associated with large language models, which rely on extensive datasets and raise the possibility of data misuse or breaches. ^6, 14^ Algorithmic bias is another significant issue, with studies cautioning that these technologies may inadvertently reinforce existing inequalities in education. ^23^

Environmental sustainability has also emerged as a serious concern in the literature. Training large GenAI models consumes enormous computational power and water resources. Training a single large model can emit more than 626,000 pounds of CO _2_, equivalent to the lifetime emissions of multiple vehicles. ^19, 31, 27^

Despite these challenges, there is growing support for a more ethically grounded, environmentally conscious use of GenAI. Institutions are being encouraged to invest in green AI technologies and incorporate sustainability education into AI-related curricula. ^8, 15^ These recommendations point toward a future where technological innovation and ethical stewardship are not mutually exclusive but mutually reinforcing.

### Identifying the gap

The literature offers a wealth of insights into the implications of GenAI in higher education, but most studies remain compartmentalized, focusing either on academic integrity, institutional policy, or ethics in isolation. What remains underdeveloped is a comprehensive framework that connects these dimensions and provides actionable guidance for educators and institutions. As AI continues to reshape academic practices, the need for such an integrated perspective becomes increasingly urgent.

This study aims to fill that gap by synthesizing existing literature across the technological, pedagogical, institutional, and ethical domains. Through a thematic review, it proposes a structured approach to understanding how universities can integrate GenAI responsibly, equitably, and sustainably in teaching and learning.

## Methods

### Research design

This study adopts a qualitative thematic literature review design to synthesize current research on the integration of GenAI in higher education. Rather than producing new empirical data, the aim is to interpret and organize existing scholarly and institutional discourse around four interconnected themes: (1) the rise of GenAI in education, (2) concerns around academic integrity, (3) institutional and policy responses, and (4) ethical and environmental implications. The purpose of this review is not only to describe these thematic trends but to critically assess them and identify a gap in the literature, specifically, the lack of an integrated framework for responsible AI adoption in higher education.

This review is conceptual and analytical, guided by the principles of thematic synthesis, ^41^ which allows for the interpretation of a diverse body of literature through the lens of meaning, significance, and practical implications.

### Literature selection strategy

The review focused on literature published between 2020 and 2025, with an emphasis on studies, policy papers, and institutional reports that directly address the impact of generative AI tools, especially ChatGPT, in university-level teaching, learning, assessment, and governance.

### Inclusion criteria

• Peer-reviewed journal articles or preprints relevant to education, educational technology, or digital ethics
• Focus on higher education or university-level contexts
• Explicit discussion of GenAI tools (e.g., ChatGPT, GPT-4, large language models)
• English-language publications
• Accessible in full text (open access or institutional subscription)

### Exclusion criteria

• Articles focused exclusively on K-12 or corporate training environments
• Non-academic opinion pieces without reference to data or scholarship
• Highly technical AI/ML studies without educational application
• Publications before 2020 (unless cited for foundational context)

A targeted keyword search was conducted using academic databases such as Google Scholar, Scopus, Web of Science, and ERIC. Search terms included combinations such as “ChatGPT in higher education,” “generative AI and academic integrity,” “AI in university teaching,” “AI and assessment,” “ethical use of AI in education,” “AI policy universities,” and “environmental impact of AI tools.” The search was limited to sources published between 2020 and mid-2025, to reflect the most recent developments in the field.

We included peer-reviewed journal articles, empirical studies, and policy papers written in English and directly relevant to higher education. To complement academic sources, we also considered institutional reports and position statements from bodies such as the Russell Group of Universities, along with select articles from major media outlets (e.g., *The New York Times*, *Reuters*, *France24*) to reflect current discourse and policy reactions.

The initial search returned over 60 sources. After screening for relevance, removing duplicates, and excluding non-substantive materials such as blogs or informal commentary, 47 core texts were selected for full-text analysis. These texts served as the foundation for the thematic synthesis presented in this study.

### Thematic analysis process

To structure the synthesis, the selected literature was subjected to manual thematic coding. Each text was read closely, and relevant passages were extracted and grouped under emergent themes using an inductive approach. The process involved:

1. Open coding of content related to GenAI’s use, challenges, and institutional responses
2. Clustering codes into broader themes (e.g., academic integrity, pedagogy, ethics, environmental impact)
3. Refining categories to ensure distinctness, internal coherence, and relevance to the research objective
4. Synthesis of insights under each theme, integrating comparative perspectives and highlighting tensions or contradictions between sources

This process was informed by thematic synthesis methodologies, which emphasize interpretive engagement over mere aggregation of findings. ^5^

### Researcher positionality

The researchers approached this study from an educational policy and digital pedagogy perspective, with a particular interest in critical digital literacy, academic ethics, and innovation in higher education. While the review strives for objectivity, thematic selection was inevitably influenced by the researchers’ academic orientation and familiarity with GenAI discourse.

### Limitations

Several limitations must be acknowledged. First, the review is limited to English-language sources, which may exclude relevant perspectives from non-English-speaking contexts. Second, while extensive, the review is not exhaustive and may have missed recent publications not yet indexed in major databases. Third, thematic synthesis, while useful for identifying patterns and insights, does not measure the frequency or empirical strength of claims in the same way as a systematic review or meta-analysis. It is also likely that the pedagogical implications of GenAI vary across disciplinary contexts, given differences in epistemic practices, assessment traditions, and acceptable forms of assistance.

Despite these limitations, this methodology enables a nuanced understanding of how GenAI is being discussed, interpreted, and responded to in the evolving context of higher education. The resulting synthesis provides a strong foundation for advancing academic policy, ethical reflection, and institutional reform. In addition, the implications of generative AI are likely to vary across disciplines, with differences in epistemic practices, assessment traditions, and acceptable forms of assistance. Future research should examine how augmented pedagogical approaches may need to be adapted for fields such as the humanities, social sciences, and STEM disciplines.

## Results

The synthesis of recent literature reveals that the integration of GenAI tools, particularly ChatGPT, into higher education is generating profound and complex transformations across pedagogical, institutional, and ethical domains. The discourse reflects both enthusiasm and anxiety: while educators and researchers acknowledge the innovative potential of these tools to enhance learning and academic operations, there is also widespread concern about their disruptive effects on academic integrity, instructional roles, and sustainable practice.

One of the most frequently cited developments is the rapid and large-scale adoption of GenAI tools by students. ChatGPT, for example, reached 300 million weekly active users by December 2024, then 400 million by February 2025, and surged to over 500 million by March–April 2025, illustrating the speed at which AI has become embedded in students’ academic practices. Numerous studies document how students are using AI to draft essays, complete assignments, summarize readings, and generate arguments and ideas in real time. ^26, 3^ These tools are no longer peripheral digital aids; they have become central components of how learners approach writing and research. Some researchers have positioned GenAI as a breakthrough for personalized learning, enabling real-time support, multilingual accessibility, and low-cost feedback loops that can enhance inclusion and flexibility in higher education. ^9, 30^ From this perspective, GenAI has the potential to democratize access to academic resources and scaffold learners who might otherwise struggle with traditional modalities.

However, this optimistic narrative is challenged by a mounting body of literature that interrogates the implications of GenAI for academic honesty and cognitive development. Students are increasingly relying on ChatGPT to produce content that can evade plagiarism detection systems. ^43, 26^ The capacity of GenAI to generate coherent, original-sounding text raises fundamental concerns about authorship, authenticity, and assessment. Instructors are now confronted with the difficult task of determining whether the work submitted by students represents their own thinking or a sophisticated prompt issued to an AI engine. ^1^ This epistemological ambiguity, ‘Am I grading the student or the machine?’, has created what some have called an “integrity vacuum” in assessment practices.

Instructors have begun voicing strong reservations. Livingstone for instance, describes resigning from teaching after observing her students’ growing dependence on ChatGPT to complete assignments. She reflects that writing is not merely about producing grammatically correct text, but about developing voice, reasoning, and identity, elements that risk erosion when students rely on machines for cognitive work. ^28^ Scholars echo this concern, warning that overreliance on AI may weaken students’ critical thinking and problem-solving skills. ^24, 29^ Rather than functioning as tools that assist deep learning, these systems may instead enable shallow engagement, particularly when students use them to bypass the intellectual struggle necessary for academic growth.

These pedagogical dilemmas are mirrored by institutional uncertainty and inconsistency. The reviewed literature shows that higher education systems remain divided in their responses to GenAI. Some institutions, such as California State University, have partnered with AI developers to pilot integration at scale, using ChatGPT for academic advising, personalized tutoring, and even administrative communication. ^33^ This proactive stance reflects a belief that institutions must adapt and modernize, integrating AI into teaching and governance in strategic and ethically informed ways. In contrast, other institutions have taken a defensive approach. The New York City Education Department blocked the use of ChatGPT on school devices, citing concerns over academic misconduct, ^13^ while Sciences Po, one of France’s most prestigious universities, imposed strict penalties for AI use, including possible expulsion. ^16^

This policy divergence exposes a deeper lack of institutional clarity. Many universities remain unprepared, lacking formal guidelines or pedagogical frameworks for GenAI use. Recent studies has called for balanced policies that inform students of acceptable AI practices while emphasizing ethical use and academic integrity. ^36^ Still, scholars argue that, in practice, faculty often navigate these changes alone, with minimal institutional support or training. ^40^ As a result, decisions about when and how students can use GenAI often vary by instructor, course, or department, leading to inconsistent experiences and growing confusion among learners. This governance vacuum signals the urgent need for sector-wide dialogue and coherent policy development.

Beyond the pedagogical and policy challenges, a fourth layer of concern emerges in the form of ethical and environmental sustainability. Multiple scholars have raised alarms about the ecological footprint of GenAI tools, which require vast computational power to operate. Training a single large-scale language model can generate over 626,000 pounds of CO _2_, comparable to the lifetime emissions of five gasoline-powered cars. ^20^ Other research further emphasize the significant water and energy usage involved in maintaining AI data centers. ^31^ These costs are often hidden from the end user, making it easy for institutions and individuals to underestimate the broader environmental impact of widespread AI deployment.

At the same time, ethical concerns about data privacy, surveillance, and bias remain salient. AI tools rely on massive datasets, some of which include unregulated or sensitive user content. Scholars warn of algorithmic bias and opaque decision-making processes that may reinforce structural inequities, particularly for marginalized learners. ^6, 14^ Meanwhile, the use of AI to monitor student behavior or generate predictive analytics raises troubling questions about autonomy and consent in educational spaces. Underfunded institutions may face particular challenges in implementing AI ethically, as the cost and complexity of integration may exacerbate existing digital divides. ^23^

Despite isolated calls for the development of sustainable AI infrastructure and responsible digital literacy initiatives, these issues remain underexamined in much of the higher education literature. Few institutional policies address the ethical lifecycle of AI systems, from training and deployment to obsolescence and e-waste. This omission suggests that higher education’s engagement with AI remains largely reactive and instrumental, focused on short-term solutions to immediate pressures rather than long-term strategic planning that considers the full spectrum of impacts.

In general, the literature reflects a convergence of pedagogical, institutional, ethical, and ecological concerns that position GenAI not simply as a tool, but as a force that is reshaping the very structure and philosophy of higher education. While the benefits of AI-assisted learning are well documented, they exist alongside serious challenges that remain insufficiently theorized or addressed at the policy level. The findings suggest a critical need for integrative frameworks that acknowledge both the promise and peril of GenAI, and that promote responsible, equitable, and sustainable practices for its adoption in higher education. These insights lay the groundwork for the discussion that follows, which explores how institutions can begin to meet this complex challenge.

## Discussion

The integration of generative AI tools such as ChatGPT into higher education is not just an incremental technological development; it signals a fundamental shift in how knowledge is produced, assessed, and valued. What emerges from the literature is not simply a clash between innovation and tradition, but a complex entanglement of promise, uncertainty, and institutional hesitation. As AI technologies become embedded in the everyday practices of students and educators, universities are being forced to confront uncomfortable questions, about the nature of learning, the integrity of assessment, and their own readiness to govern emerging digital norms. At the same time, some studies suggest that students are not uniformly uncritical adopters of AI, but actively negotiate when and how these tools align with their own learning goals and ethical judgments. These findings resonate with prior research demonstrating that learning processes integrating cognitive, affective, and behavioral dimensions are more likely to result in sustained engagement and action. ^38^

Across the reviewed literature, it is clear that students are adapting quickly. ChatGPT has become a routine companion in academic work, often used to draft essays, clarify concepts, or even generate argument structures. Qualitative and survey-based research suggests that students actively negotiate ethical boundaries, credibility, and learning value when deciding how and when to use AI tools, indicating that student engagement with generative AI is not uniformly uncritical or instrumental. ^42^ Large-scale sector evidence reinforces this pattern. A national survey of higher education students indicates that the use of generative AI for academic tasks, including assessed work, has become widespread, while institutional guidance remains uneven and, in many cases, unclear. ^21^ This widespread use has outpaced institutional frameworks, many of which are either silent on the matter or offer contradictory guidance. Educators, caught between unfamiliar tools and mounting concerns over plagiarism and grade validity, are left to improvise. Several scholars point to the deep tension here: when AI-generated texts can bypass traditional detection tools, and when students can use these tools to produce competent writing with minimal engagement, what does academic work actually represent ^43, 1^? The very notion of authorship begins to blur. This aligns with emerging efforts to move beyond reactive approaches to AI misuse, emphasizing the need for pedagogical strategies that actively engage students in ethical and critical uses of generative technologies. ^39^

Recent sector-level discussions increasingly emphasize a shift away from detection-oriented responses to generative AI toward approaches grounded in transparency and process-based assessment. Rather than attempting to define fixed thresholds of acceptable AI use, these perspectives foreground understanding how student work is produced over time, positioning academic integrity as a pedagogical and relational practice rather than a purely technological problem.

This tension has real consequences. Some educators, like Victoria Livingstone, have stepped away from teaching altogether, disillusioned by the erosion of intellectual labour in the classroom. For her, and for many others, writing is not simply a skill but a mode of thinking, a space where students develop their analytical voice. If that process is outsourced to a machine, what are we left with ^28^? This concern resonates throughout the literature. We risk raising a generation of students fluent in prompting but not in reasoning, trained to manipulate outputs rather than interrogate ideas. ^24, 29^

And yet, the impulse to respond with outright bans seems shortsighted. Several institutions, such as Sciences Po in Paris or the New York City Education Department, have taken hard stances against AI tools. ^13, 16^ While understandable, these approaches may deepen the divide between institutions that engage critically with AI and those that suppress its presence entirely. Banning AI can inadvertently create educational inequities, disadvantaging students who are denied access to tools that others are being taught to use responsibly. ^12, 34^

Across higher education, there is growing acknowledgement that institutional policy development has lagged behind students’ rapid adoption of generative AI, contributing to uncertainty for educators and learners alike. This misalignment reinforces calls for adaptive, dialogic policy frameworks that evolve alongside pedagogical practice rather than relying on static or prohibition-based rules.

The literature points instead toward a more thoughtful middle ground. What is needed is not prohibition, but integration with boundaries. Universities must invest in clear, transparent policies that differentiate between acceptable and unacceptable uses of GenAI. But policy alone is not enough. There is also a pedagogical task at hand: to reimagine assessment in ways that privilege process over product, originality over polish, and live performance over polished automation. Oral exams, in-class reflections, scaffolded assignments, and collaborative projects all offer ways to assess what students can actually do and think, rather than what AI can generate for them. ^35^

Contemporary debates increasingly frame the challenge of generative AI in education not in terms of determining how much AI use is “too much,” but in identifying where, when, and why cognitive responsibility should remain with learners within the learning process. This perspective aligns with pedagogical approaches that emphasize reflection, transparency, and guided use of AI as integral to learning design.

This discussion also reveals a larger shift in the role of educators. No longer mere transmitters of knowledge, faculty are now expected to serve as ethical guides, digital mentors, and facilitators of judgment in an age of abundant automation. ^17, 25^ Yet most educators have not been trained for this transition, and professional development remains sparse. Without systemic support, universities risk placing a transformative burden on individuals who are already stretched thin.

Equally pressing are the ethical and environmental dimensions of GenAI’s rise. Despite being less visible in day-to-day academic life, the carbon footprint of AI development is significant. The training and maintenance of large language models require vast amounts of energy and water, contributing to environmental degradation that remains largely absent from institutional AI policies. ^19, 31^ Data privacy, algorithmic bias, and the opacity of AI decision-making also loom large, especially in under-resourced institutions where ethical safeguards may be weaker. ^6, 14^

What becomes clear through this analysis is that the challenges posed by GenAI cannot be addressed in isolation. They are pedagogical, institutional, ethical, and ecological all at once. This means the response must also be multidimensional. Universities need coherent, flexible frameworks that support experimentation while safeguarding core academic values. Students need digital and ethical literacy, not just access. And educators need space to rethink their roles, not just new rules to follow.

In many ways, higher education is standing at a crossroads. The choices institutions make now, about how to integrate, regulate, and educate around AI, will shape not only what learning looks like in the years ahead, but also what it means. The task is not to reject technology, nor to accept it uncritically, but to shape it deliberately. That will require courage, collaboration, and a willingness to confront difficult questions about what we expect of learners and of ourselves.

## Conclusion

The rise of generative artificial intelligence in higher education, particularly tools like ChatGPT, has brought institutions face-to-face with a new pedagogical reality, one that challenges long-held assumptions about authorship, learning, and assessment. This paper has explored how GenAI is not only transforming the mechanics of student work but reshaping the very foundations of academic practice. From student behavior and institutional policy to questions of ethics, equity, and environmental responsibility, the impact of AI is wide-ranging and undeniably profound.

What emerges from this thematic review is the urgent need for a balanced, strategic, and values-driven response. Banning AI tools outright may offer short-term control, but it ignores the broader cultural and technological shift already underway. Similarly, embracing AI without ethical guardrails risks undermining the integrity and purpose of education itself. The answer lies somewhere in between: in deliberate, transparent integration that enhances human learning rather than replaces it; in assessment models that emphasize originality and depth of understanding; and in policies that educate rather than punish.

This paper contributes to the growing discourse by proposing a multidimensional framework for thinking about GenAI in education, one that considers not just the immediate practical challenges, but also the deeper institutional, ethical, and ecological implications. In doing so, it advocates for what might be called *augmented pedagogy*: a vision of education where AI supports, but does not supplant, the role of the teacher; where students use tools critically, not blindly; and where learning remains anchored in inquiry, interaction, and integrity.

Unlike substitution-oriented models of educational technology adoption, augmented pedagogy explicitly foregrounds human cognitive agency, ethical responsibility, and institutional accountability as non-delegable elements of the learning process.

In practical terms, augmented pedagogy can be understood as an approach in which generative AI is deliberately positioned as a scaffold within learning processes rather than as a shortcut to outcomes. Future empirical research is needed to examine how augmented pedagogical approaches operate in practice across disciplines, assessment formats, and institutional contexts, particularly in relation to student learning outcomes and equity. This includes designing tasks that require students to document, reflect on, and justify their use of AI tools, as well as assessments that make reasoning, iteration, and decision-making visible. Such an approach shifts the pedagogical focus from controlling AI use to cultivating informed, accountable engagement with it.

As the higher education sector adapts to this shift, three priorities stand out. First, institutions must urgently revise their assessment systems to reflect the new realities of AI-enhanced authorship. Second, they must invest in the digital and ethical literacy of both students and faculty, ensuring that AI is used responsibly and inclusively. And third, they must address the hidden environmental and social costs of these technologies by embedding sustainability into AI policy and practice.

Ultimately, the integration of GenAI into academia is not a question of if, but how. The challenge now is to shape that integration wisely, protecting the core values of higher education while preparing learners for a future where critical thinking, ethical judgment, and human creativity matter more than ever. The stakes are high, but so is the opportunity: to reimagine a system of learning that is not just compatible with AI, but made stronger, more just, and more meaningful because of it.

## Ethics and consent

This study is based exclusively on the analysis of published literature and publicly available documents. No human participants, personal data, or identifiable information were involved. Therefore, ethical approval and participant consent were not required for this study.

## Data Availability

No primary data were generated or analyzed in this study. The research is based exclusively on the analysis of published literature and publicly available documents. All sources used in the analysis are cited within the article and listed in the reference section. No extended data are associated with this article.
