# Supplementary material for: Generative AI in education: Process-aware pedagogy, assessment integrity, and institutional governance
Source: Open Res Eur. 2026 May 13;6:161. [Version 1] doi: 10.12688/openreseurope.23343.1 (PMC13276483; doi:10.12688/openreseurope.23343.1)
Supplement: Peer review history

# Reviewer report: Reviewer response for version 1

**DOI:** 10.21956/openreseurope.25254.r75150

**Published:** 2026-06-17

**Authors:** Laura Trujillo-Liñán

**Affiliations:** 1 Universidad Panamericana, Mexico City, Mexico City, Mexico

The article is interesting and addresses current and important issues related to the development, discussion, and critical reflection on Generative AI (GenAI) and the role of universities. The author(s) clearly state from the outset the objectives of the article, the methodology, the scope and limitations of the study, and the proposed approach. These elements are presented early in the manuscript and appear appropriate and understandable.

 However, the author(s) indicate that, based on their research process, 60 sources were initially identified, of which 47 were ultimately included in the study. It would be important to specify which sources were selected and, if possible, to provide a more detailed account of the findings derived from each source, including appropriate citations with year and page numbers. The author(s) also mention that relevant literature published between 2020 and 2025 was reviewed; however, it is not possible for the reader to determine which specific sources were analyzed.

 Upon reviewing the reference list, I noticed that some cited works appear to be absent from the discussion, including references to authors affiliated with institutions such as MIT and Stanford. Furthermore, several arguments and examples are repeated throughout the manuscript without substantial development. For instance, the example of a professor who stopped teaching after observing how students were using GenAI appears at least four times, yet no additional details are provided regarding the individual, the institution, the context, or comparable experiences from other universities.

 As a result, the manuscript lacks the depth that could be achieved through a more detailed analysis of concrete cases. I would encourage the author(s) to include specific examples, identify the institutions involved where appropriate, describe the circumstances of each case, and provide a more thorough interpretation grounded either in the literature reviewed or in their own analytical framework.

 Additionally, I do not find a sufficiently original or clearly articulated proposal emerging from the study. The manuscript points out that tools such as ChatGPT are increasingly being used and that universities have not yet fully adapted to these developments; however, this observation has already been widely discussed in the literature. The paper would benefit from offering a more distinctive contribution, deeper insights, or novel findings derived from the reviewed studies.

 Overall, I believe the manuscript would be strengthened by incorporating more detailed evidence from the literature, presenting concrete case studies, and developing a deeper and more original analysis of the implications of GenAI for higher education.

Is the study design appropriate and does the work have academic merit?

Partly

Is the work clearly and accurately presented and does it cite the current literature?

Partly

If applicable, is the statistical analysis and its interpretation appropriate?

Not applicable

Are all the source data underlying the results available to ensure full reproducibility?

Yes

Are the conclusions drawn adequately supported by the results?

Partly

Are sufficient details of methods and analysis provided to allow replication by others?

Partly

Reviewer Expertise:

AI, Ethics, Philosophy, Higher Education, Online Education, Media, Pedagogy.

I confirm that I have read this submission and believe that I have an appropriate level of expertise to confirm that it is of an acceptable scientific standard, however I have significant reservations, as outlined above.

# Reviewer report: Reviewer response for version 1

**DOI:** 10.21956/openreseurope.25254.r74253

**Published:** 2026-06-10

**Authors:** Zouhaier Slimi

**Affiliations:** 1 National University of Sciences and Technology, Sohar, Oman

The article addresses a timely and relevant topic: generative AI in higher education, focusing on process-aware pedagogy, assessment integrity, institutional governance, and ethical sustainability.

1. Clarity and current literature: Partly. The article is clearly written and cites recent literature on GenAI, ChatGPT, assessment, institutional policy, and AI ethics. However, the evidence base is uneven because peer-reviewed studies, institutional reports, media sources, and policy commentary are sometimes treated together without sufficient distinction.
2. Study design and academic merit: Partly. A qualitative thematic literature review is appropriate for this conceptual topic, and the article has academic merit. However, the review type needs clearer justification. The article should explain more explicitly whether it is a narrative review, thematic review, scoping review, or conceptual synthesis.
3. Methods and analysis: Partly. The article states that more than 60 sources were initially identified and that 47 core texts were selected for full-text analysis. However, the search and screening process is not sufficiently reproducible. The authors should provide exact search dates, database-specific search strings, the number of records retrieved from each database, details on duplicate removal, exclusion criteria, and, preferably, a PRISMA-style flow diagram.
4. Thematic synthesis: Partly. The article identifies four main themes: GenAI adoption, academic integrity, institutional responses, and ethical/environmental implications. However, the coding process is insufficiently detailed. The authors should add a table showing examples of initial codes, theme development, and how the 47 sources contributed to each theme.
5. Statistical analysis: Not applicable. The article does not include quantitative data or statistical testing.
6. Source data and reproducibility: Partly. The article states that no primary data were generated and that all analysed sources are cited. However, in a literature review, reproducibility still requires a transparent list of sources and a coding record. The authors should provide an appendix or extended data file listing the 47 included texts, their type, inclusion rationale, and thematic allocation.
7. Conclusions: Partly. The conclusions are generally consistent with the article’s discussion, especially the call for augmented pedagogy, transparent AI use, process-based assessment, and institutional governance. However, some claims are broad and should be more carefully qualified, especially claims about student dependence, erosion of critical thinking, and environmental impacts.
8. Points that must be addressed for scientific soundness:

- Provide a reproducible literature search and screening protocol.
- Add a complete table of the 47 included sources.
- Clarify the type of review and justify the methodological approach.
- Explain the coding and thematic synthesis process in more detail.
- Distinguish peer-reviewed evidence from media, policy, and institutional sources.
- Revise the data availability statement to include the review corpus and coding matrix.
- Moderate broad claims so that conclusions remain fully supported by the reviewed evidence.

Overall, the article is promising and relevant, but it requires methodological strengthening before it can be considered fully scientifically sound.

Is the study design appropriate and does the work have academic merit?

Partly

Is the work clearly and accurately presented and does it cite the current literature?

Partly

If applicable, is the statistical analysis and its interpretation appropriate?

Not applicable

Are all the source data underlying the results available to ensure full reproducibility?

Partly

Are the conclusions drawn adequately supported by the results?

Partly

Are sufficient details of methods and analysis provided to allow replication by others?

Partly

Reviewer Expertise:

AI in education, AI in TESOL, AI in research

I confirm that I have read this submission and believe that I have an appropriate level of expertise to confirm that it is of an acceptable scientific standard, however I have significant reservations, as outlined above.

# Reviewer report: Reviewer response for version 1

**DOI:** 10.21956/openreseurope.25254.r75149

**Published:** 2026-06-06

**Authors:** Teuta Agaj Avdiu

**Affiliations:** 1 University of Gjilan, GJILAN, Kosovo (Serbia and Montenegro)

This manuscript addresses one of the most timely and consequential developments currently shaping higher education: the rapid integration of generative artificial intelligence into teaching, learning, assessment, and institutional governance. The paper provides a comprehensive and well-structured thematic review of recent literature and successfully synthesizes a broad range of scholarly, policy, and institutional perspectives. A particular strength of the manuscript is its balanced approach. Rather than adopting either a celebratory or alarmist position, the authors critically examine both the opportunities and challenges associated with generative AI, including academic integrity, assessment practices, institutional preparedness, ethical considerations, and environmental sustainability.

 The analysis is thoughtful, coherent, and appropriately grounded in the literature reviewed. The authors move beyond a simple summary of existing studies and identify important tensions and gaps that characterize current debates. The interpretation of findings is convincing and demonstrates a strong understanding of the complexity of AI integration in higher education. Importantly, the conclusions are well supported by the evidence presented and follow logically from the thematic synthesis.

 The manuscript makes a valuable contribution through its proposal of an “augmented pedagogy” approach, which positions AI as a support for learning rather than a substitute for human cognition and intellectual engagement. This framework has considerable potential and could become an important contribution to the field. To strengthen the paper further, the authors may wish to elaborate this concept in greater detail, clarify its relationship to existing pedagogical frameworks, and provide a more explicit conceptual model. Additionally, minor enhancements to the methodological description, including greater transparency regarding the literature selection process, would improve rigor. Overall, this is a strong, relevant, and highly indexable manuscript that contributes meaningfully to ongoing discussions about the future of higher education in the age of AI.

Is the study design appropriate and does the work have academic merit?

Yes

Is the work clearly and accurately presented and does it cite the current literature?

Yes

If applicable, is the statistical analysis and its interpretation appropriate?

Not applicable

Are all the source data underlying the results available to ensure full reproducibility?

Yes

Are the conclusions drawn adequately supported by the results?

Yes

Are sufficient details of methods and analysis provided to allow replication by others?

Yes

Reviewer Expertise:

Teaching

I confirm that I have read this submission and believe that I have an appropriate level of expertise to confirm that it is of an acceptable scientific standard.
